# Supplementary material for: Different structural variant prediction tools yield considerably different results in Caenorhabditis elegans
Source: PLoS One. 2022 Dec 30;17(12):e0278424. doi: 10.1371/journal.pone.0278424 (PMC9803319; doi:10.1371/journal.pone.0278424)
Supplement: Attachment — Submitted filename: lesack_response_to_reviews_08-Nov-22.pdf [file pone.0278424.s015.pdf]

We thank each of the reviewers for the time that they spent reviewing the manuscript. We have sought to address their comments in the manuscript, providing clarification where necessary, both in the manuscript and our responses below.

Editor

Acknowledgments Section: We have removed the funding information from the latest draft of the manuscript. The information given in the submission portal is correct.

Data Availability statement: We have updated the data availability statement in the submission portal. It is not feasible to make all the data available (it runs into terabytes). However, we have created a tutorial that allows any interested reader to recreate our simulated data. This is included on the GitHub repository.

Two table 3s: This is now fixed in the submitted manuscript.

SOM tables S1 to S13: These tables are now separated and uploaded.

Reviewer 1

Reviewer's comment 1: The interpretation of results in real data needs to be substantially revised. As no benchmark is available for real data, we could not use regular recall/precision/F1 score to assess SV discovery accuracy. Some alternative approaches should be used to compare the performance of SV callers, instead of simply showing low consistency between SV callers. There is much more work needed to be done after this point. The significance of this manuscript is largely determined by how SV callers are evaluated in real data without benchmark datasets. What the authors have presented are far from sufficient to answer the questions raised by authors at the beginning.

Authors' response: On reflection, we agree that we incorrectly use the term benchmark when referring to real-world data and did not use recall/precision/F1 scores. We do not make claims as to the accuracy of the callers on real-world data. Our original plan had been to use the real PacBio data as a gold standard set for benchmarking. However, we were surprised to discover a high level of inconsistency between long-read SV callers. This led us to benchmark the long-read callers with simulated data. The only expert curated data for *C. elegans* is from Maroilley *et al.*, which is used (also see our response to Comment 5 below). We have changed the manuscript text to make it clear where we are benchmarking with simulated data and where we are comparing the predictions of SV callers with real-world data. We had already discussed that benchmarking against real data is so far only possible with human-specific resources, such as the 1000 Genome Project.

Reviewer's comment 2: In the abstract, authors stated "multiple short-read and long-read tools were benchmarked using real and simulated data". I would suggest revising such statements in the manuscript, as it is usually not considered as "benchmarked" in real data if no reference SV callset is available.

Authors' response: As in our response above, we agree with this comment and have changed the manuscript to distinguish between benchmarking and comparing predictions.

Reviewer's comment 3: In the Introduction section, authors claimed that there are few public long-read datasets for non-human organisms. In fact, although benchmark datasets are relatively rare for non-human species, samples sequenced by both short-read and long-read platforms are pretty common in SRA. There are also projects like Vertebrate Genomes Project that really focuses on non-human organisms. I understand that C. elegans is the focus of this paper, but it may limit the audiences to C. elegans researchers, as benchmark results in C. elegans cannot represent other species if authors claim that benchmark in human cannot represent C. elegans.
Authors' response: Here, we presume that the reviewer is referring to the following sentence: *At time of writing, most curated, large, multi-population, whole genome datasets for metazoan species are restricted to humans.* We apologise if this statement is confusing. The key phrase here is 'multi-population'. For this work, we need several distinct isolates of a given species that have both short- and long-read data, with the associated metadata. At the time we started this work, we found only *C. elegans* fit our criteria; we had looked at the VGP. Given the reviewer's comments, we revisited the VGP. We went through the SRA data for the VGP umbrella project (https://www.ncbi.nlm.nih.gov/bioproject/489243) and identified 762 genomic sequencing runs from 33 species (there were other species with just transcriptome data under this project). From all the metadata we could not find a species where multiple, clearly defined isolates had been sequenced. We anticipate the flood of these population WGS data in the next few years. In addition to the VGP, we looked at cattle sequencing data and discovered that some of our concerns have been raised in cattle (https://www.sciencedirect.com/science/article/pii/S0022030217303521). The authors show little overlap between short- and long-read SV predictions, though do not explore these trends as deeply as we do. We agree that we can only present a *C. elegans*-centric view. However, we believe that the results of our study, given the robustness of the *C. elegans* data, will encourage people to use SV callers with caution whether using invertebrate or non-human vertebrates in the future.

Reviewer's comment 4: In Table 3, it is quite surprising that long-read SV callers showed such low recall for duplications, except for Sniffles. Majority of the simulated SVs are shorter than 10kbp and should be reported by long reads. Duplications are sometimes considered as insertions by some SV callers. I know Sniffles, pbsv, and SVIM do report insertions in the VCF file. How were the insertion calls treated? If an insertion event is reported at a true duplication site with same SV size, should this duplication still be considered as FN?
Authors' response: We were also surprised. Further, it is an understandable assumption that "Majority of the simulated SVs are shorter than 10kbp and should be reported by long reads." However, it is not what we see when using the long-read SV callers. The reviewer makes an excellent observation that "[d]uplications are sometimes considered as insertions." We do not seek to treat them as special cases for a couple of reasons. First, our motivation here is to show what a typical researcher would see when running these software. Second, each of the longread callers is capable that we evaluated is capable of discriminating between novel sequence insertions and tandem duplications. If interspersed duplications are present, the duplicated sequence could in theory be categorized as insertions by callers whose duplication calls are limited to those that occur in tandem. Of the longread callers that were evaluated, only SVIM is able to predict interspersed duplications. Duplications misclassified as insertions could be potentially be identified by mapping the inserted sequences against the reference genome, however, this goes beyond what the typical analyses that a typical researcher would do when

using this software. We also excluded any interspersed duplications predicted by SVIM, as this SV type was not supported by any other caller.

We have revised the manuscript to describe our reasons for excluding insertions and to clarify that the duplication category only includes tandem duplications.

Reviewers' comment 5: In the section of 'prediction of known structural variants', only three previously validated SVs were used for benchmark short-read SV callers. Could the authors clarify if there are only three SVs in the benchmark dataset, or did they select just one SV from each SV type? Based on these three SVs in one sample, if they are not clinically relevant or extremely hard to identify, the results in this section are not significant enough to demonstrate which SV callers are more accurate, considering the fact that there are hundreds of SVs per C. elegans genome in last table.
Authors' response: In response to the recommendation of a previous reviewer, we sought a dataset of validated SVs. To the best of our knowledge, Maroilley *et al.* is the only in *C. elegans* where predicted SVs have undergone expert review. From this study, we removed short SVs (<50bp) or complex SVs (inverted tandem duplications). We accept that it is a limited survey, but we consider it informative for the reader.

Reviewer's comment 6: The use of Jaccard index to represent SV calling accuracy could be a novel approach. According to its definition and its applications in simulation benchmarks, the Jaccard index could be biased to large SVs as they contain more numbers of base pairs. One thing we can try is to set a maximal allowed SV size when calculating Jaccard index. For example, if we set the cutoff at 2kbp, we only count 2000 bp for all SVs longer than the cutoff, so that a single 4Mbp FP will not reduce the Jaccard index a lot.
Author's response: Our use of the Jaccard index was a focus on recommendations by an earlier reviewer and we made changes to the manuscript submitted to PLOS One. We have previously considered setting a maximal allowed SV size, but any cut-off will always be arbitrary. However, it response to the above comment, we have elaborated on the strengths and weaknesses of using the Jaccard index in the methods, results, and discussion sections.

Reviewer's comment 7: For the simulation benchmark in results section, I would suggest adding some more details about how mock genomes were generated, especially the number of SVs per genome. It would help readers who are not very familiar with C. elegans genome.
Authors' response: We agree with the recommendation and have included more information in the manuscript.

Reviewer's comment 8 (minor): Why was Manta not included in the short-read SV caller comparison? It is also a widely used SV caller. In our previous experience of SV discovery in human genomes, Manta often performs better than other SV callers.
Authors' response: As with any benchmarking/comparison paper, it is almost impossible to capture all methods. One paper describes 69 different tools. We selected short-read SV predictors that were readily compatible with SVE/FusorSV pipeline, which does not yet include Manta. We also considered the most cited methods: DELLY (1556 citations), BreakDancer (1511 citations), CNVnator (1409 citations), and LUMPY (1074 citations). By comparison, Manta has 1031 citations at present (October 21st, 2022). While on par with LUMPY, feasibility

meant that we had to draw the line somewhere. In the discussion, we now state explicitly that our comparison is not exhaustive.

Reviewer's comment 9 (minor): In the methods section, parentheses should be used in equations for Precision and Recall calculation. Precision = TP / (TP + FP) and Recall = TP / (TP+FN).
Authors' response: Thank you for pointing out this mistake, it has been fixed.

Reviewer's comment 10 (minor): There are two 'Table 3' in the manuscript.
Authors' response: Thank you for pointing out this mistake, it has been fixed.

<u>Reviewer 2</u>
Reviewer's comment: I appreciate the authors attention to the revisions and think this will be cited as a methodological caution to the field. I am glad to see the paper come out to the field. I have nothing more to add.
Authors' response: We thank the reviewer for taking the time to review the manuscript for a second time and for considering our revisions to previous reviews.

<u>Reviewer 3</u>
Reviewer's comment (we assume minor): Perhaps a missing part of the discussion is the fact that some recent approaches for structural variant calling rely on the combination and consensus of multiple tools (eg. Parliment2, Zarate et al 2020 Gigascience, which is briefly mentioned in the introduction, but the overall concept is not revisited).
Authors' response: We thank Dr. Doyle for all his positive comments on the manuscript. With regard to the one above, we agree that a fuller discussion on combination approaches. We have elaborated on this in an improved discussion.
